# Diminished reactivity effect of confidence rating on perceptual decision-making in depression

**DOI:** 10.3389/fnins.2025.1638734

**Published:** 2025-09-10

**Authors:** Luoya Zhang, Yelu Liu, Yuxiang Wang, Ke Gong, Kezhi Liu, Wei Lei, Jing Chen

**Affiliations:** ^1^Department of Psychiatry, The Affiliated Hospital of Southwest Medical University, Luzhou, China; ^2^School of Clinical Medicine, Southwest Medical University, Luzhou, China; ^3^Fundamental and Clinical Research on Mental Disorders Key Laboratory of Luzhou City, Luzhou, China

**Keywords:** confidence ratings, confidence reactivity effect, metacognition, drift diffusion model, metacognitive control, metacognitive monitoring

## Abstract

**Introduction:**

Retrospective confidence ratings (CRs) after decision-making reactively lead to prolonged response times (RTs) and improved decision accuracy, a phenomenon known as the reactivity effect. This effect reflects an individual’s metacognitive control processes. Little is known if depressive pathologies modify the reactivity effect in patients with Major Depressive Disorder (MDD).

**Methods:**

This study investigated the differences in the reactivity effect between 94 patients with MDD and 97 healthy controls (HCs), using a perceptual decision-making task and the Drift Diffusion Model (DDM) analysis.

**Results and discussion:**

The results demonstrated that prompted CRs significantly prolonged RTs in both groups. However, prompted CRs improved decision accuracy in HCs, this effect was absent in the MDD group. DDM analysis revealed increased decision thresholds under CR conditions for both groups. Crucially, a significant group×condition interaction emerged for drift rate (v), with HCs demonstrating enhanced evidence accumulation speed compared to MDD patients. These findings indicate impaired metacognitive reactivity effects in MDD through confidence monitoring, highlighting deficits in metacognitive monitoring and control processes associated with depression.

## 1 Introduction

Major depressive disorder (MDD) is a prevalent and burdensome condition characterized by persistent depressed mood, diminished motivation or anhedonia, as well as physical and cognitive symptoms (WHO, 2023). Metacognition, defined as “knowledge of one’s cognitive processes” (Flavell, 1979), including the introspective ability to monitor and control one’s cognitive processes effectively, is essential for regulating human behavior, decision-making, problem-solving and overall well-being (Jang et al., 2020; Seow et al., 2021). Metacognitive dysfunction represents a core feature of MDD pathophysiology (Hoven et al., 2019; Sun et al., 2017). Clinical evidence consistently indicated that impaired metacognitive abilities were associated with both maladaptive behavioral patterns and reduced quality of life across various neurological and psychiatric disorders (Hoven et al., 2019; Seow et al., 2021; Sun et al., 2017).

Metacognitive functions are usefully divided into monitoring and control processes. Metacognitive monitoring refers to the ability to appraise one’s knowledge, proficiency, or task performance (Flavell, 1979), with confidence ratings serving as a primary metric for assessing performance monitoring and subjective accuracy perception. In experimental paradigms, participants typically make perceptual decisions (first-order judgments) followed by retrospective confidence evaluations regarding decision accuracy. These confidence ratings enable quantification of metacognitive ability through assessing the associations between decision accuracy and task performance (Fleming and Lau, 2014). Accumulating evidence suggests that impairments in metacognitive processes are closely linked to various psychopathological conditions (Drueke et al., 2023; Hoven et al., 2019; Sun et al., 2017), particularly depression. More specifically, confidence bias involving metacognitive monitoring, defined as the tendency for an individual to report persistently high or low confidence levels irrespective of the actual accuracy (Fleming and Lau, 2014), is associated with biased evaluation and detrimental decision-making. Many depressed patients exhibited negative confidence bias in both prospective and retrospective confidence judgments across multiple cognitive domains, including memory, general knowledge, perceptual discrimination, and social judgment tasks, demonstrating predictive validity for depressive symptom maintenance (Drueke et al., 2023; Moses-Payne et al., 2019). Notably, Hong et al. (2025) found that the negative metacognitive bias in MDD patients is characterized by a tendency to report low levels of confidence regardless of their actual task performance, and this bias is significantly correlated with the severity of depressive symptoms. These findings confirm that negative confidence bias represents a core feature of anxious-depressive symptomology, with such metacognitive monitoring abnormalities potentially perpetuating emotional dysregulation through maladaptive cognitive patterns like perseverative thinking (LeMoult and Gotlib, 2019).

Additionally, metacognitive control encompasses the process of behavioral modifications guided by metacognitive monitoring to achieve a desired cognitive goal based on information hypothetically acquired (e.g., decision confidence; Flavell, 1979). The process of eliciting confidence ratings requires continuous self-monitoring and provides crucial insights into cognitive functions across metacognition, perception, and memory domains (Double and Birney, 2024). Recent evidence suggested that eliciting confidence judgments from participants may induce *reactivity* effects on task performance (Bonder and Gopher, 2019; Double and Birney, 2017, 2018; Lei et al., 2020; Li et al., 2024; Mitchum et al., 2016), specifically measurable alterations in decision response times (RTs) or decision accuracy. This phenomenon, known as the reactivity effect of confidence ratings, has been mainly examined in retrospective confidence ratings across numerous cognitive domains, particularly in memory tasks (Mitchum et al., 2016), perceptual decision-making (Baranski and Petrusic, 2001; Petrusic and Baranski, 2003), reasoning and problem-solving (Double and Birney, 2017, 2018). For instance, Petrusic and Baranski found that requiring confidence ratings increased decision RTs in a sensory discrimination task (Petrusic and Baranski, 2003), suggesting an additional computational burden due to the need for accurate confidence judgments, specifically the need to monitor performance in order to make accurate confidence judgments (Double and Birney, 2024). Notably, this process not only extended RTs but also enhanced accuracy as individuals engage in thorough information processing and error-monitoring mechanisms (Li et al., 2024; Yeung and Summerfield, 2012). Our previous study demonstrated that providing confidence ratings significantly prolonged decision RTs and improved decision accuracy in healthy population (Lei et al., 2020), and these findings were confirmed recently (Li et al., 2024). In addition, a positive reactivity effect was observed in recognition memory task, particularly those relying on item memory (Zhao et al., 2023). Meta-analysis further revealed modest positive effects on free recall and medium-to-large positive effects on recognition (Zhao et al., 2023). Confidence ratings may provoke participants’ feelings of uncertainty about decision accuracy, prompting them to gather more information or evidence, thereby consuming additional cognitive resources (Li et al., 2024). The cognitive psychology model posits that confidence reporting activates dual mechanisms: bottom-up monitoring of decision evidence and top-down allocation of cognitive resources, constituting an integrated metacognitive control system (Narens, 1990). Hence, the reactivity effect represents a direct influence of metacognitive monitoring on online cognitive processing, i.e., metacognitive control. Through metacognitive monitoring and control, the metacognitive ability impacts diverse cognitive functions. However, the effects of confidence rating on decision accuracy remain inconsistent across experimental paradigms (Ackerman et al., 2020; Baranski and Petrusic, 2001; Double and Birney, 2017, 2018, 2019; Petrusic and Baranski, 2000). Thus, further research is required to confirm the effect of confidence ratings on decision accuracy.

Clinical studies have consistently demonstrated that individuals with MDD exhibited a negative confidence bias, indicative of deficit metacognitive monitoring (Hoven et al., 2019). Within the cognitive process, executive function (EF) demonstrates close associations with metacognition. Specifically, error detection and effort monitoring are correlated with metacognitive monitoring, while error correction, inhibitory control, and resource allocation are related to metacognitive control (Fernandez-Duque et al., 2000). Previous studies have revealed significant deficits in both executive function and attention among patients with depression compared to healthy controls (Nuño et al., 2021; Snyder, 2013). EF and metacognition are conceptualized as higher-order cognitive processes that enable individuals to operate flexibly and adapt efficiently to new and challenging tasks. Deficits in these processes may directly compromise the metacognitive control mechanisms responsible for regulating and adapting cognitive strategies during decision-making. Impairments in this area make it more challenging for individuals with MDD to make complex decisions in dynamic environments, affecting their ability to adjust decisions based on environmental feedback. Therefore, it is conjectured that abnormalities in metacognition may influence the decision-making of depressive patients. However, it remains unclear whether individuals with MDD exhibit abnormalities in metacognitive control processes, particularly the reactivity effects that enable real-time behavioral adjustments based on ongoing performance evaluation.

The reactivity effect is typically assessed by comparing RTs and decision accuracy between conditions where participants provide prompted confidence ratings (DCR+) versus conditions without such ratings (DCR-) during decision-making process. However, direct comparisons of RTs or accuracy alone cannot elucidate how elicited confidence ratings modulate the computational architecture of decision-making processes. A promising approach for investigating the reactivity effect impairments in depressive patients involves deconstructing the components of the decision-making process. The drift-diffusion model (DDM) is a sequential sampling model well-suited for explaining the computational processes involved in simple two-alternative decision processes (Ratcliff, 1978). DDM assumes that decisions are made through a noisy process accumulating information over time from a starting point toward one of two response criteria or boundaries. DDM characterizes decision-making processes using four parameters: the rate of evidence accumulation (drift rate, v), the amount of evidence required to reach a decision (decision threshold, a), a bias toward one of the two response options (bias, z), the amount of time not dedicated to the decision-making process (non-decision time, t). Previous studies have demonstrated that depressive patients exhibited reduced drift rates and elevated decision thresholds (Lawlor et al., 2020; Pitliya et al., 2022), suggesting slower evidence accumulation and more conservative response strategies compared to healthy controls. Research on confidence reactivity in healthy populations demonstrated that confidence ratings resulted in improved decision accuracy, prolonged RTs, and increased decision thresholds, consistent with increased conservatism in evidence accumulation (Li et al., 2024). In depression, diminished confidence levels may exacerbate decision hesitancy and information-seeking tendencies, potentially manifesting as exaggerated threshold elevation during confidence reporting conditions. Thus, using DDM could effectively dissect how reactivity effects differentially manifest in the decision circuitry of MDD patients through quantitative characterization of evidence accumulation dynamics.

This study aimed to investigate the reactivity effect in patients with MDD. We hypothesized that reactivity effect may be impaired in MDD patients, potentially manifesting as (1) longer mean RTs without corresponding improvements in decision accuracy (contrasting with typical CR-induced enhancements observed in healthy populations), and (2) altered DDM parameters, such as elevated response thresholds or compromised evidence accumulation efficiency.

## 2 Methods

### 2.1 Participants

Ninety-four patients with MDD (mean age: 17.25 ± 2.09; range: 14–25 years; 63 women, 31 men) were recruited from the Psychiatry Department of the Affiliated Hospital of Southwest Medical University (Luzhou, China). The sample included a higher proportion of women (67%) due to the greater number of female patients in the ward, which may reflect the higher prevalence of MDD in women (Li et al., 2023). Patients were clinically diagnosed with depression using ICD-10 criteria, and their diagnosis of MDD was reconfirmed by two trained psychiatrists using MINI International Neuropsychiatric Interview Chinese version 5.0 (MINI; Sheehan et al., 1998). Ninety-seven healthy controls (HCs; mean age: 17.72 ± 2.09; range: 15–24 years; 74 women, 23 men) were recruited from local communities and schools through advertisements on social media. HCs were excluded if they had any current or past psychiatric diagnosis, as assessed using the MINI. All participants were Han Chinese, had normal or corrected-to-normal vision, no self-reported history of color blindness or color weakness, and were right-handed. Exclusion criteria for both groups included severe physical diseases, history of substance or alcohol abuse/dependence, accepted electroconvulsive therapy, and history of head trauma with loss of consciousness. Written informed consent was obtained from all participants, and for those participants under 18 years old, consent was also obtained from their guardians. Study procedures were fully explained, including the risks and benefits, and the voluntary nature of participation. The study protocol was approved by the Research Ethics Committee of the Affiliated Hospital of Southwest Medical University (No. KY2020222) and was carried out in accordance with the Declaration of Helsinki.

### 2.2 Scales

All participants provided demographic information. Depression and anxiety symptoms were assessed using self-report measures in the past week, including the Beck Depression Inventory (BDI; Beck et al., 1961) and the Beck-Anxiety Inventory (BAI; Beck et al., 1988) for all participants. The self-esteem scale (SES) was used to measure subjects’ overall feelings about self-worth and self-acceptance (Rosenberg, 1965), where a higher score indicates a higher level of self-esteem.

### 2.3 Experimental design and procedure

#### 2.3.1 Experimental procedure

**Figure 1:**
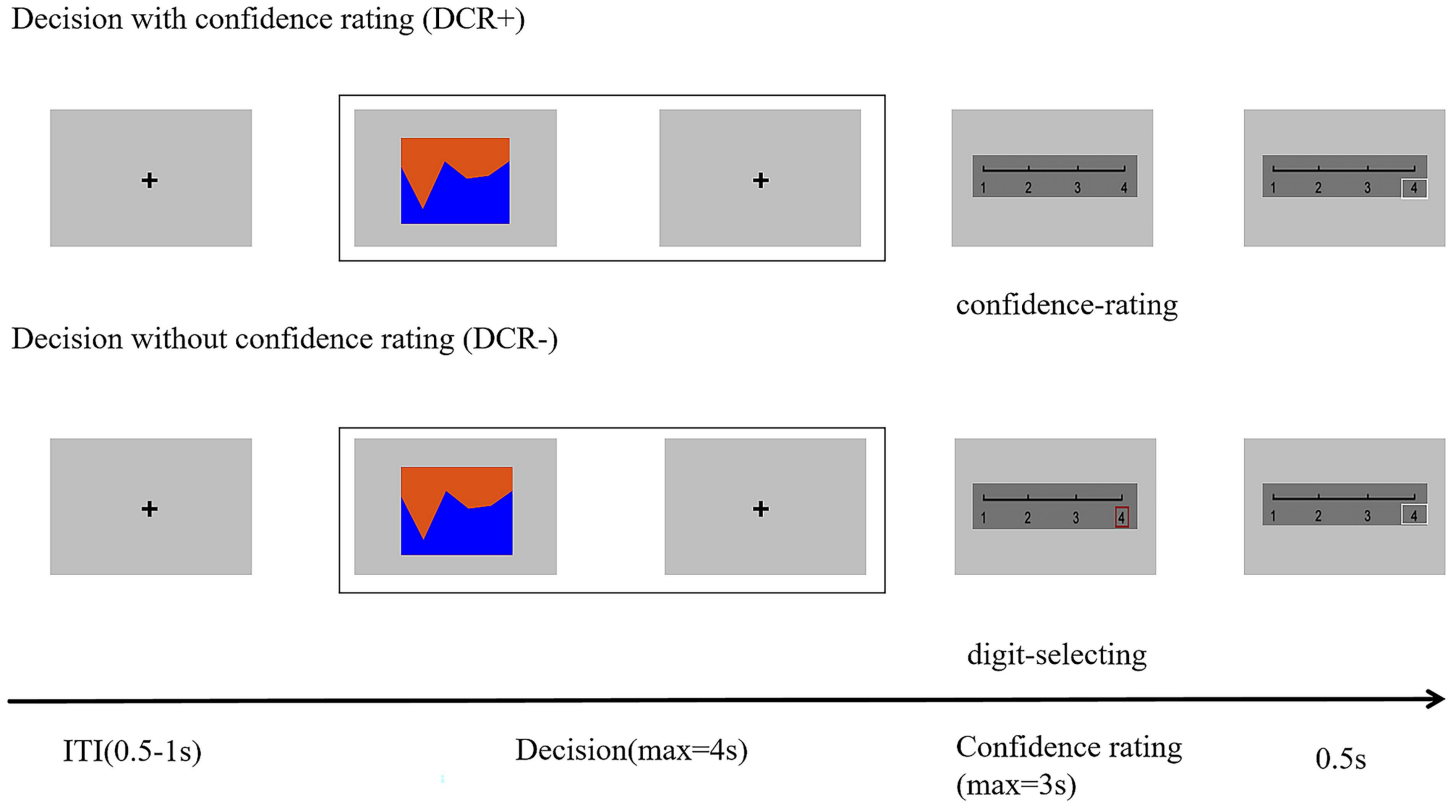
Experimental procedure of the task. Each trial began with a fixation cross (ITI) lasting between 0.5–1 s randomly. This was followed by a rectangular picture stimulus displayed at the center of the screen, divided into two sections of different colors (orange and blue). Participants were required to identify which color occupied a larger area by pressing “1” or “2” within 4 s. After area judgment, participants either indicated their confidence in the correctness of their decision (confidence rating) or selected a randomly highlighted number (digit selection) within 3 s. DCR+, Decisions with confidence rating; DCR-, Decisions without confidence rating.

The experiment employed a within-subjects block-design using E-prime 2.0 (Verdonschot et al., 2019). There were two types of blocks, namely perceptual decision-making with retrospective confidence rating (DCR+) or without retrospective confidence rating (DCR-), as illustrated in Figure 1.

In the DCR + condition, each trial began with an inter-trial interval (ITI) of 0.5–1 s, followed by a rectangular picture stimulus displayed at the center of the screen, divided into two areas with different colors (orange/blue). Subjects were required to determine which color occupied the greater area by pressing “1” for the top area or “2” for the bottom area within 4 s as quickly as possible. The stimulus disappeared upon response, and a fixation cross would present for the remainder of the 4 s. Participants then rated their confidence in the accuracy of their decision on a scale from 1 (lowest confidence) to 4 (highest confidence) within 3 s, using the number keys 1–4 on the keyboard. The selected number would be framed in a white box for 0.5 s after the rating. The DCR- condition was identical to DCR+, except that instead of a confidence rating, participants were required to press a number key (1–4) that was randomly marked out by the computer. Any trials where participants did not make a response within the designated response windows (4 s for area judgment and 3 s for confidence rating/number selection) were excluded from the analysis.

Before the formal experiment, 20 trials were practiced to familiarize participants with the task. Based on previous research (Baranski and Petrusic, 2001), the task contained 200 trials, divided into four blocks of 50 trials each. The order of the two block types (DCR + and DCR-) was counterbalanced between subjects, i.e., for half of the subjects, the block order was DCR+, DCR-, DCR+, and DCR-, and reversed for the other half of the subjects. At the beginning of each block, the experiment informed subjects whether they would make confidence ratings or number selections.

In the decision stage, a correct response earned 1 point, while incorrect responses received zero points. No feedback was given during the task, but the total earned points would be displayed on the screen at the end of the task. Participants received financial compensation, with extra bonus according to the total earned points (100 points = ¥20). Participants were told that their compensation would depend on the points they earned and that they should try to earn as many points as possible, but they were not informed of the exact calculation method.

#### 2.3.2 Stimuli materials

Perceptual stimuli were created by splitting a rectangle (199 * 150 pixels) into two areas (filled by either orange or blue) with a random jagged line (6 variable points), using MATLAB 2016a. The principal stimuli consisted of 100 pictures selected from a pool of 800, informed by a pilot study with an independent group of subjects (*N* = 52). The chosen pictures demonstrated an area-judging accuracy of approximately 70% (accuracy ranged from 55.77 to 78.85%, mean accuracy: 68.96%; area difference ranged from −1.999 to 1.989 units). Note that the units were determined arbitrarily but applied consistently across all stimuli. Importantly, the same set of stimuli was used for all participants and conditions. That is, for each participant, the same 100 pictures were present twice, once in the DCR + condition and once in the DCR- condition. This design aimed to loosely maintain task difficulty around the 70% accuracy level established in the pilot study. The color locations were counterbalanced, with 50 pictures showing orange at the top and blue at the bottom, while the other 50 reversed this.

### 2.4 Data analysis

#### 2.4.1 Clinical and demographic measures

Clinical and demographic data were compared using t-tests and chi-square tests as appropriate. Participants’ performance was measured using decision accuracy and RTs, with RTs defined as the interval from stimulus presentation to motor time. Based on a previous study, trials with RTs less than 150 ms and more than 2,500 ms were excluded for DDM analysis (Lawlor et al., 2020).

#### 2.4.2 Task data analysis

RTs and accuracy were calculated separately for the DCR + and DCR- conditions. Behavioral data were analyzed using a 2 (groups: MDD patients and HCs) × 2 (experimental conditions: DCR + and DCR-) repeated-measures ANOVA in SPSS 26.0, with group as a between-subjects factor and experimental condition as a within-subjects factor. The reactivity effect was characterized by two indices, ∆accuracy and ∆RTs, using the following (Equations 1, 2):


(1) Δaccuracy=accuracy(DCR+)−accuracy(DCR−).

(2) ΔRTs=RTs(DCR+)−RTs(DCR−).

#### 2.4.3 Metacognitive metrics

We calculated three quantitative measures of metacognition, namely metacognitive sensitivity (meta-d’), metacognitive criterion (meta-c), and mean confidence (also referred to as metacognitive bias). Meta-d’ quantifies the extent to which confidence ratings distinguish between correct and incorrect judgments and is also known as metacognitive accuracy. Meta-c can be interpreted as the confidence criteria over an alternative model from which the subject is constructing their confidence (Sherman et al., 2018). Lastly, the mean confidence reflects the tendency for an individual to report a high or low confidence level irrespective of the actual accuracy (Fleming and Lau, 2014). The meta-d’ was computed according to the method and script introduced by Fleming and Lau (Fleming and Lau, 2014). The meta-c was calculated using an equation based on (Sherman et al., 2018):


$$\text{Meta}-c=(c^{'}/d^{'})*\text{meta}-d^{'}$$

Where c' and d' stand for criteria and discrimination index in first-level decision-making.

#### 2.4.4 DDM analysis

The Hierarchical Bayesian estimation of the Drift-Diffusion Model toolbox (HDDM) in the Jupyter Notebooks environment (Wiecki et al., 2013) was used for modeling. HDDM employed a Markov chain Monte Carlo (MCMC) sampling method to estimate posterior parameter distributions. The model parameters included decision threshold (a), drift rate (v), and non-decision time (t). Based on previous studies, there was no bias between response options that have an equal chance of being correct. Therefore, the starting point was at the midpoint between the two boundaries (Stafford et al., 2020).

Four plausible models were evaluated to ascertain the best fit for the observed data. Model fit was assessed by comparing the models’ Deviance Information Criteria (DIC), with lower DIC values indicating a preferable fit. For each model, three MCMC chains were performed, containing 50,000 samples, with 5,000 samples per chain discarded as burn-in. Convergence was assessed by plotting and visually inspecting traces and autocorrelation plots for each estimated parameter. Furthermore, the values of Gelman and Rubin were calculated, which should be close to 1 and not exceed 1.02 if the chains have converged successfully (Wiecki et al., 2013). The final model choice was based on a combination of model fit and convergence.

To assess reactivity effects, we compared posterior distributions of the parameters between groups and conditions using HDDM. A significant difference between conditions (DCR + vs. DCR-) indicates a significant reactivity effect. To further illustrate group differences in reactivity effects, we extracted the estimated parameter values and analyzed them using a Two-factor ANOVA, with condition (DCR + vs. DCR-) as a within-subject factor, and group (MDD vs. HC) as a between-subject factor. A significant group-by-condition interaction indicates a significant group difference in reactivity effects.

#### 2.4.5 Linear mixed-effects models

While the ANOVAs provide a focused test of our a priori hypotheses concerning group and condition effects on reactivity effect, this approach does not account for potential confounding factors that may influence the decision-making processes of participants. To explore how clinical symptoms (BDI, BAI, SES), metacognitive metrics (meta-d’, meta-c, mean confidence), and DDM parameters (a, v, t) contribute to participants’ decision-making, we constructed two linear mixed-effects models (LMMs) predicting decision accuracy and RTs separately, using these variables as predictors, while allowing the intercept to be varied among participants (Subject):

We used group, condition, scale scores, DDM parameters, and metacognitive metrics as predictors, and allowed the intercept to be varied among participants (Subject):


$$\begin{array}{l} \mathrm{DV}\sim {\text{group}}^{*}\text{condition}+\mathrm{SES}+\mathrm{BDI}+\mathrm{BAI}+a+v+t\\ +\text{meta}-d^{'}+\text{meta}-c+\text{mean confidence}+(1\;|\;\text{Subject}) \end{array}$$

Where DV can be either decision accuracy or decision RTs.

We fitted these models using the brms package in R (Bürkner, 2017) with 4 chains, 6,000 iterations per chain, and 3,000 warmup iterations.

## 3 Results

### 3.1 Sample characteristics

Table 1 shows group differences in demographics and scale measures. No significant differences were found between MDD patients and HCs in age (*p* = 0.118) and sex (*p* = 0. 155). However, the MDD patients showed higher BDI and BAI scores, but lower SES scores compared to HCs, indicating more severe depressive and anxiety symptoms and decreased general self-esteem in patients. Furthermore, correlation analysis revealed a marginally significant negative correlation between SES scores and BDI scores (*r* = −0.202, *p* = 0.051) in MDD patients, but not in HCs (*r* = 0.091, *p* = 0.377), suggesting an association between depressive symptoms and self-esteem in MDD.

**Table 1:** Demographic and clinical characteristics of the participants.

Variables	MDD patients (*n* = 94)	Healthy controls (*n* = 97)	t/χ^2^	*p*
Sex (male/female)	94(31/63)	97(23/74)	2.02	0.155
Age	17.25 ± 2.09	17.72 ± 2.09	−1.57	0.118
BDI	31.33 ± 10.61	6.23 ± 6.99	19.373	<0.001^***^
BAI	25.88 ± 13.37	4.27 ± 7.19	13.979	<0.001^***^
SES	25.05 ± 3.00	26.15 ± 2.05	−2.974	0.003^**^
Mean confidence	2.76 ± 0.56	3.11 ± 0.46	−4.736	<0.001^***^
Meta-d’	1.14 ± 0.48	1.12 ± 0.45	0.203	0.839
Meta-c	0.07 ± 0.35	0.05 ± 0.31	0.266	0.791

^**^p < 0.01; ^***^p < 0.001. MDD, Major Depressive Disorder; BDI, Beck Depression Inventory; BAI, Beck Anxiety Inventory; SES, The self-esteem scale; Meta-d’, metacognitive sensitivity; Meta-c, metacognitive criterion.

Regarding metacognitive metrics, MDD patients showed lower mean confidence than HCs. No significant group differences were found in Meta-d’ and meta-c (Table 1).

### 3.2 Reactivity effect in RTs and decision accuracy

First, independent samples *t*-tests were conducted to examine the potential sequence influences of the DCR + and DCR- conditions. The results suggested that no significant sequential effect in the experiment (*p* > 0.05). Based on this, the data from both experimental sequences were combined for overall statistical analysis. All results are shown in Table 2.

**Table 2:** Behavioral data and DDM results of different groups.

Parameters	MDD patients (*n* = 94)		Healthy (*n* = 97)		Main effect of the experimental condition			Main effect of the group			Interaction effect		
	DCR+	DCR-	DCR+	DCR-	F	*p*	partial *η*^2^	F	*p*	partial *η*^2^	F	*p*	partial *η*^2^
Decision accuracy	0.721 ± 0.084	0.711 ± 0.091	0.749 ± 0.083	0.720 ± 0.098	20.968	<0.001	0.100	2.214	0.138	0.012	5.453	0.021	0.028
Decision RTs	1315.086 ± 296.845	1243.018 ± 276.883	1331.165 ± 268.854	1285.812 ± 243.890	40.758	<0.001	0.177	0.591	0.443	0.003	2.110	0.148	0.011
decision threshold	1.687 ± 0.226	1.595 ± 0.242	1.730 ± 0.221	1.623 ± 0.234	86.680	<0.001	0.314	1.286	0.258	0.007	0.442	0.507	0.002
drift rate	0.626 ± 0.190	0.633 ± 0.210	0.705 ± 0.192	0.647 ± 0.218	6.682	0.010	0.034	2.902	0.090	0.015	11.149	0.001	0.056
non-decision time	0.685 ± 0.197	0.673 ± 0.172	0.686 ± 0.174	0.704 ± 0.154	0.095	0.759	0.000	0.457	0.500	0.002	2.287	0.132	0.012

MDD, Major Depressive Disorder; DCR+, Decisions with confidence rating; DCR-, Decisions without confidence rating; RTs, Response times.

For mean RTs, there was a significant main effect of condition (*p* < 0.001). RTs were significantly prolonged under the DCR + condition in both groups. However, neither the interaction effect between the condition and group nor the main effect of group (*ps* > 0.05) was significant.

And for decision accuracy, there was a significant condition-by-group interaction effect (*p* = 0.021). Pair-wise comparisons showed that decision accuracy in the HC group was significantly higher under the DCR + condition compared to the DCR- condition (0.749 ± 0.083 vs. 0.720 ± 0.098, *t* = 4.884, *p* < 0.001, Cohen’s d = 0.526). Conversely, this condition difference in decision accuracy was not observed in the MDD group (*p* > 0.05). The main effect of the experimental condition was significant (*p* < 0.001), whereas the main effect of the group was not significant (*p* > 0.05).

**Figure 2:**
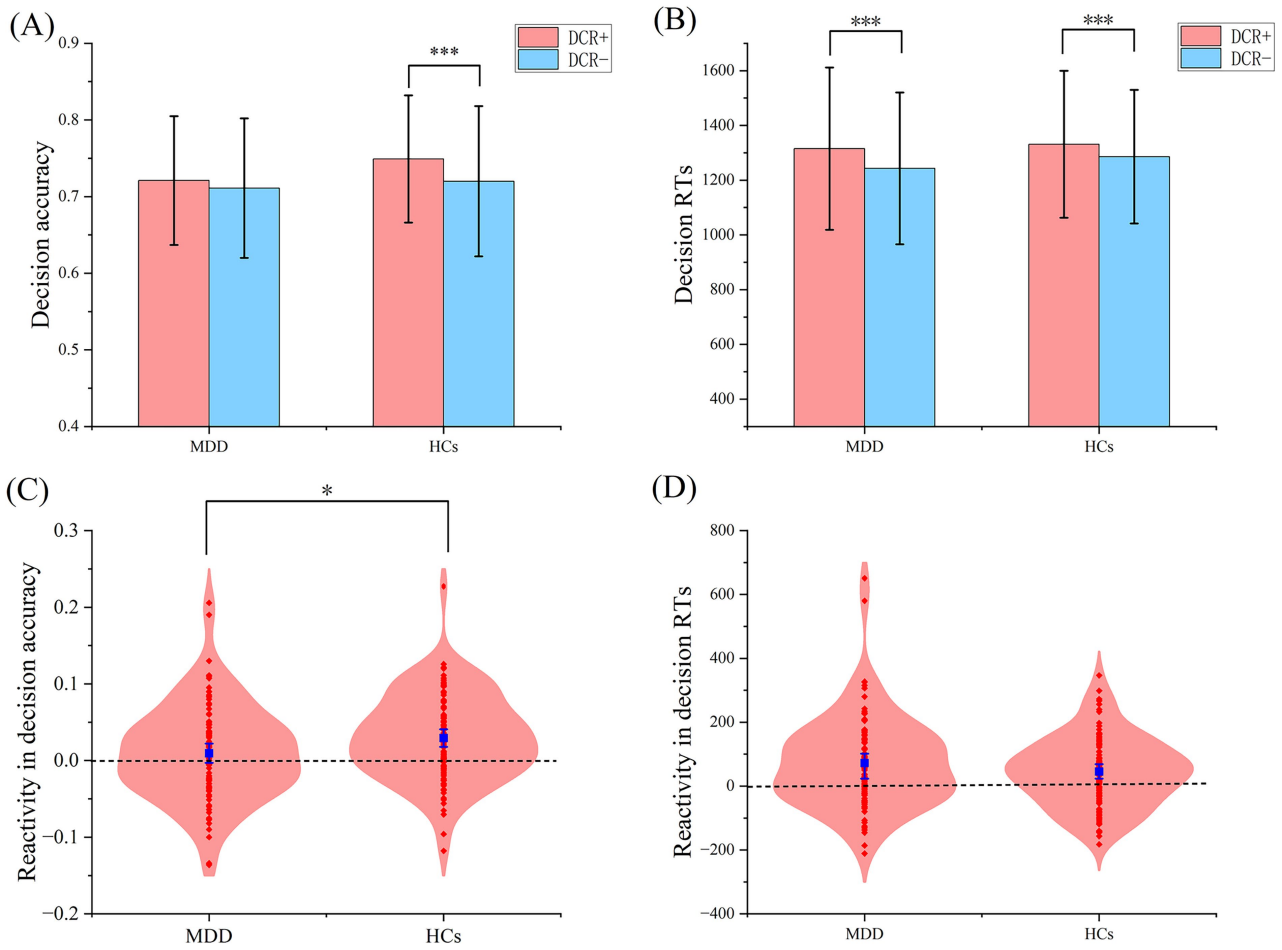
Reactivity effect in RTs and decision accuracy Comparisons of decision accuracy **(A)** and RTs **(B)** between the DCR + and the DCR- in different groups. The violin plots **(C,D)** depicted the distributions of the reactivity effect of confidence ratings (i.e., the difference in decision accuracy and RTs between the DCR + and DCR-). Each red dot represented one participant’s reactivity effect and the blue points represented group averages. Error bars indicated the 95% confidence intervals. RTs, Response times; DCR+, Decisions with confidence rating; DCR-, Decisions without confidence rating. MDD, Major Depressive Disorder patients; HCs, healthy controls.

Similarly, the difference in ∆accuracy between the two groups was significant (0.010 ± 0.061 vs. 0.029 ± 0.056, *t* = −2.335, *p* = 0.021, Cohen’s d = −0.336), but the ∆RT demonstrated no significant difference (72.069 ± 143.056 vs. 45.353 ± 109.397, *t* = 1.453, *p* = 0.148, Cohen’s d = 0.210; see Figure 2).

### 3.3 Differences in confidence level between groups

The present study compared mean confidence levels and confidence levels during accurate and inaccurate decisions between different groups. Results indicated that, regardless of decision accuracy, depressed patients consistently displayed significantly lower confidence levels than HCs (2.778 ± 0.562 vs. 3.132 ± 0.458, *t* = −4.778, *p* < 0.001, Cohen’s d = −0.687). Specifically, when decisions were correct, confidence levels in MDD patients were notably lower than HCs (2.693 ± 0.469 vs. 2.852 ± 0.556, *t* = −2.133, *p* = 0.034, Cohen’s d = −0.309). This significant difference persisted even during incorrect decisions (2.482 ± 0.428 vs. 2.693 ± 0.473, *t* = −3.223, *p* = 0.001, Cohen’s d = −0.467).

### 3.4 DDM results

We further tested four models to examine three specifications of DDM. First, we incorporated all three parameters (decision threshold “a,” drift rate “v,” and non-decision time “t”) into the model for fitting, resulting in a model Deviance Information Criterion (DIC) value of 82142.45. Subsequently, we fitted models containing one parameter at a time: the model with only parameter “a” had a DIC value of 133952.38, the model with only parameter “v” had a DIC value of 140666.28, and the model with only parameter “t” had a DIC value of 100099.18. Therefore, the model incorporating all three parameters best fitted the data. The Gelman-Rubin value of the final model was 1.003, and chains and autocorrelations confirmed adequate convergence for all parameters.

**Figure 3:**
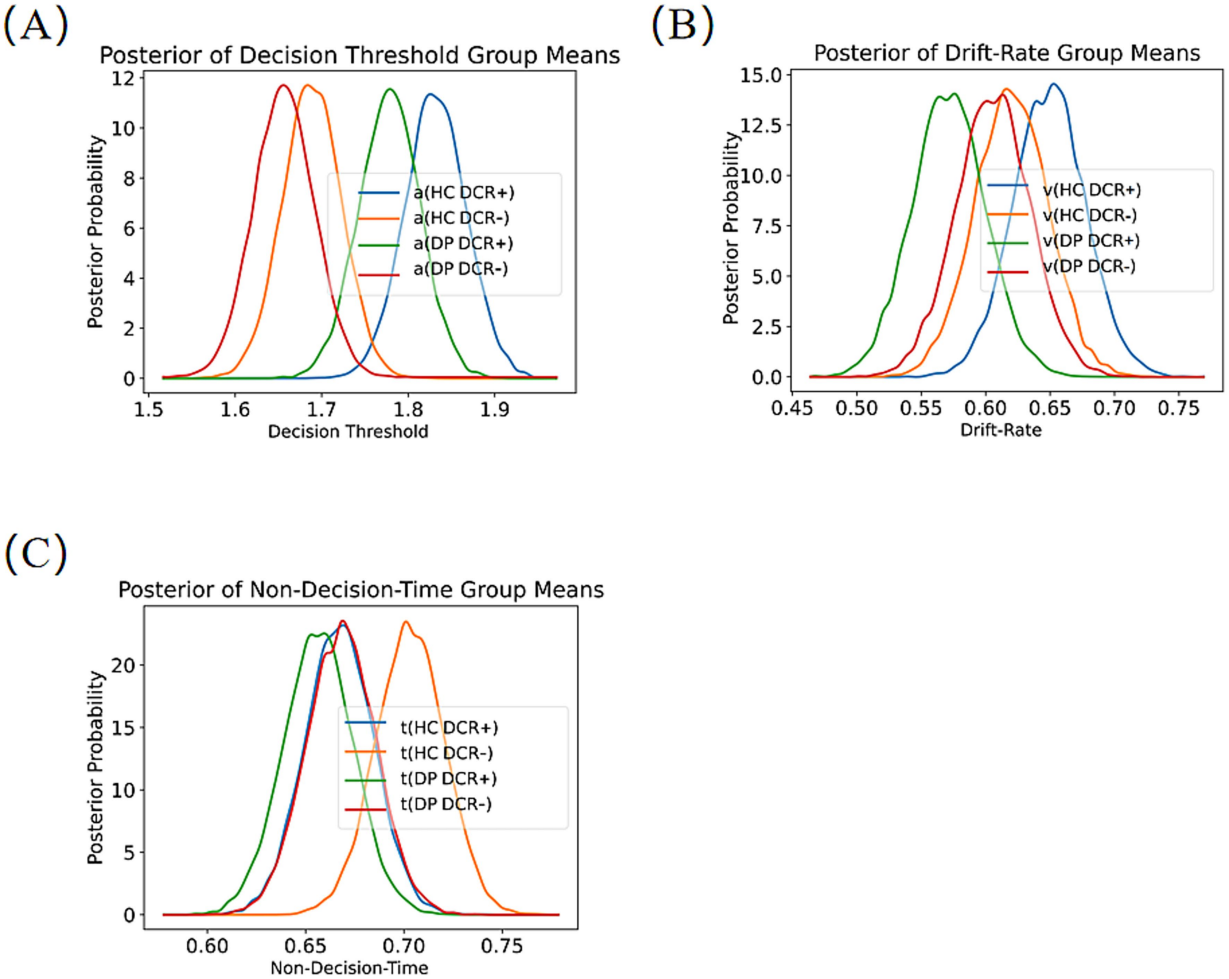
Posterior distributions of DDM parameters Comparisons of the posterior distributions of the parameters between the DCR + and the DCR- in different groups. DCR+, Decisions with confidence rating; DCR-, Decisions without confidence rating. HC, Healthy controls, DP, Depressive patients.

We compared the posterior distributions of the parameters using HDDM (Figure 3). For the decision threshold (a), the decision threshold was higher under DCR + condition than under DCR- condition for both groups (*p* < 0.001). No significant group differences were presented in both conditions (*p* > 0.14). Further two-way ANOVA showed no significant group-by-condition interaction.

Regarding the drift rate (v), in DRC + condition, we observed a lower v in MDD than in HCs (*p* < 0.027); no significant group differences were noticed in the DCR- condition (*p* = 0.364). No significant condition differences were found in both groups (*p* > 0.229). Further two-way ANOVA revealed a significant group-by-condition interaction, where *v* was significantly higher under DCR + condition compared to the DCR- condition in the HCs (0.705 ± 0.192 vs. 0.647 ± 0.218, *t* = 4.323, *p* < 0.001, Cohen’s d = 0.441), but not in the MDD patients (*p* > 0.05).

For the non-decision time (t), no significant group or condition differences were found (*p* > 0.068). Two-way ANOVA revealed no significant effects.

### 3.5 Results of mixed linear models

The mixed linear regression showed that decision accuracy can be significantly predicted by meta-d’, meta-c, v, and a (Table 3). Additionally, in line with the findings on group difference in reactivity effect, we also noticed that the group-by-condition interaction effect was significantly predictive of decision accuracy. For the RTs, the DDM parameters (v, a, and t) were significantly predictive of decision RTs. No scale scores (SES, BDI, and BAI) were significantly predictive of either ACC or RT. These results indicated that metacognitive metrics and DDM parameters are associated with decision accuracy and its reactivity effects, while only DDM parameters were associated with the variability of decision RTs.

**Table 3:** Fixed effect coefficients of linear mixed models.

Predictors	Estimate	Est. Error	l–95% CI	u-95% CI
DV = ACC				
Intercept	0.000	0.007	−0.014	0.014
Group	−0.021	0.013	−0.046	0.004
Condition	−0.017	0.005	**−0.027**	**−0.007**
SES	0.006	0.007	−0.008	0.021
BDI	0.001	0.016	−0.030	0.031
BAI	0.012	0.013	−0.013	0.036
v	0.901	0.010	**0.881**	**0.920**
a	0.327	0.007	**0.313**	**0.342**
t	0.004	0.007	−0.010	0.018
meta_d’	0.032	0.011	**0.011**	**0.053**
meta_c	−0.019	0.008	**−0.034**	**−0.004**
Mean confidence	0.001	0.008	−0.015	0.018
Group*condition	−0.022	0.005	**−0.031**	**−0.012**
DV = Decision RT				
Intercept	0.000	0.005	−0.010	0.010
Group	0.007	0.009	−0.011	0.025
Condition	−0.007	0.004	−0.015	0.000
SES	0.004	0.006	−0.007	0.014
BDI	0.004	0.011	−0.018	0.026
BAI	0.012	0.009	−0.006	0.029
v	−0.123	0.007	**−0.137**	**−0.109**
a	0.605	0.005	**0.594**	**0.616**
t	0.605	0.005	**0.594**	**0.615**
meta_d’	0.005	0.008	−0.011	0.020
meta_c	0.008	0.006	−0.003	0.019
Mean confidence	−0.004	0.006	−0.016	0.007
Group*condition	0.002	0.004	−0.005	0.009

Statistically significant effects (95%CI not including zero) were highlighted with bold fonts. DV, Dependent Variable; ACC, Accuracy; RT, Reaction Time; SES, self-esteem scale; BDI, Beck Depression Inventory; BAI, Beck Anxiety Inventory; v/a/t(DCR+), drift rate/decision boundary/non-decision time under DCR+ condition; Meta-d’, metacognitive sensitivity; meta-c, metacognitive criterion.

## 4 Discussion

This research examined the effects of confidence ratings requirements on the decision-making behaviors in individuals with MDD, utilizing a post-decision confidence rating task. Our results demonstrated that prompted retrospective trial-by-trial confidence ratings in the DCR + condition resulted in significantly longer RTs for both groups compared to the DCR- condition. While HCs exhibited improved decision accuracy under DCR + relative to DCR-, this enhancement was absent in MDD patients. The DDM analyses further indicated that both groups had elevated decision threshold under the DCR + condition relative to the DCR- condition. More importantly, HCs displayed increased drift rate (v) under the DCR + condition (vs. the DCR- condition), which was not exhibited in MDD patients.

This study discovered that post-decision confidence ratings alter cognitive performance by prolonging RTs in both MDD and HC groups. Consistent with previous studies, providing confidence ratings can extend the decision RTs (Baranski and Petrusic, 2001; Lei et al., 2020; Li et al., 2024; Petrusic and Baranski, 2003). Utilizing the DDM, this study further revealed that decision thresholds increased in both groups under the DCR + condition compared to the DCR- condition. These findings aligned with the increased conservatism theory (Li et al., 2024), which posits the continuous requirement for confidence ratings concerning decision judgments may heighten participants’ uncertainty regarding the accuracy of those decisions. Specifically, uncertainty about whether a recent decision was correct may prompt participants to re-examine the evidence or seek a second opinion (Banca et al., 2015). This increased conservatism theory predicts that reporting confidence should lengthen decision RTs through increasing the boundary threshold (Beste et al., 2018; Stafford et al., 2020). In line with this perspective, participants may raise their decision threshold to gather more information before making a decision, ensuring their decisions are sufficiently accurate to maintain or enhance their confidence levels under the DCR + condition. The results of LMMs supported this view by demonstrating that decision threshold positively, while draft rate and non-decision time negatively, predicted decision RTs and accuracy in decision-making. That is, a more conservative decision process (bigger *a* value but smaller *v* and *t* value) leads to more accurate but slower responses, maybe through requiring more information before making a decision (Ratcliff and McKoon, 2008). Note that, condition did not significantly (95%CI: [−0.015, 0.000]) predict RT in the LMM model, but was significant in the ANOVA. The discrepancy may be accounted for by the fact that LMM model considered the effect of DDM parameters (Ratcliff and McKoon, 2008). Furthermore, prompted confidence ratings could add an additional computational burden, which competes for cognitive resources needed to make the primary decision, resulting in longer decision time (Double and Birney, 2024). This could explain the effects observed in decision threshold and non-decision time. Taken together, prompted retrospective trial-by-trial confidence ratings demonstrated the reactivity effect of RTs in both MDD and HC groups.

More importantly, the present study revealed that providing post-decision confidence ratings significantly enhanced the decision accuracy only in the HCs, aligning with the previous findings (Bonder and Gopher, 2019; Lei et al., 2020; Li et al., 2024). Significant group-by-condition interaction have been observed in both ANOVAs and LMM analysis, suggesting a robust effect despite adjusting for covariates. For instance, judgments of learning (JOLs) have been shown to have a positive reactivity effect on memory performance in word-list learning paradigms (Li et al., 2021; Shi et al., 2023; Zhao et al., 2022). The prolonged RTs and increased decision accuracy observed in our finding suggested that making retrospective metacognitive ratings may enhance engagement during perceptual decision tasks (Shi et al., 2023), consistent with the cognitive benefit hypothesis positing that confidence ratings confer some benefits to performance monitoring, facilitating cognitive processes such as more effective response strategies, rule-learning or reallocation of cognitive resources, leading to better performance (Double and Birney, 2017). Bonder et al. also argued that the reactivity was an inevitable consequence of the metacognitive monitoring process established during the task formation phase (Bonder and Gopher, 2019). In cognitive neuroscience, metacognitive monitoring is defined as the awareness individuals have of their own cognitive processes and their ability to monitor and reflect on them (Flavell, 1979). In the present study, these prompted retrospective trial-by-trial confidence ratings can be considered a form of metacognitive monitoring. Metacognitive control refers to an individual’s self-regulatory mechanisms, such as planning and adapting behavior based on such monitoring (Flavell, 1979). Thus, the reactivity effect of decision RTs and decision accuracy can be considered as indices of metacognitive control.

However, the absence of reactivity effect on accuracy in MDD patients contrasts with the performance enhancement observed in HCs under confidence rating conditions. Substantial evidence has been found for a decline in confidence in depression, indicating a negative confidence bias that reveals abnormal metacognitive monitoring (Drueke et al., 2023; Hoven et al., 2019). Recent research indicated that people with greater subclinical anxious-depression symptoms have global underconfidence, which may stem from impaired integration between local and global metacognitive processes (Katyal et al., 2025). This phenomenon was consistent with our findings and may be related to the lower self-confidence and self-esteem characterizing MDD patients compared to HCs. Reduced self-esteem heightens vulnerability to negative feedback and rumination, potentially driving impaired self-evaluation and confidence erosion (Sowislo and Orth, 2013). Notably, reasoning task studies have shown that confidence ratings enhanced performance only in individuals with high baseline self-confidence, while potentially impairing those with low confidence thresholds (Double and Birney, 2017, 2019). In our study, MDD patients exhibited significantly lower average confidence levels than HCs, irrespective of the comparable task performance. In addition, substantial evidence indicated that confidence ratings integrated multimodal information sources, including past experience with similar decisions (Boldt et al., 2019) and global beliefs about one’s competence (Rouault and Fleming, 2020). In MDD, these processes may generate aberrant evaluations of decision quality, potentially triggering task-irrelevant processing, anxiety, and self-doubt that compromise metacognitive control mechanisms. Previous study also suggested that the difficulties depressed individuals face with decision-making largely result from their failure to use effective decision-making techniques (Leykin et al., 2011). Moreover, cognitive control difficulties interact with cognitive biases to hinder cognitive switching, working memory updating, and inhibition of irrelevant information (Villalobos et al., 2021). This cognitive profile manifested behaviorally through prolonged response times and reduced decision accuracy in MDD patients, collectively indicating metacognitive control deficits. Our LMM analysis showed that metacognitive sensitivity positively predicted decision accuracy. A higher metacognitive sensitivity, a better alignment between subjective confidence and objective task performance (Fleming and Lau, 2014), indicates a better metacognitive monitoring function. This enhanced monitoring could facilitate decision-making through mechanisms such as modulated resource allocation and exploration (Boureau et al., 2015), cognitive offloading (Risko and Gilbert, 2016) and changes in evidence processing and enhanced readout of post-decisional evidence (Stone et al., 2025) and eventually greater decision accuracy. The group-by-condition interaction suggested that confidence ratings differentially affect decision-making in healthy controls and MDD individuals, even after accounting for clinical symptoms and DDM parameters. Consequently, the prompted retrospective trial-by-trial confidence ratings may have different reactivity effect on decision accuracy, with MDD patients potentially unable to effectively utilize metacognitive evaluations for performance optimization.

Furthermore, metacognition demonstrates substantial theoretical and neurobiological overlap with executive function (EF), particularly in developmental progression and associated brain networks (Fernandez-Duque et al., 2000; Roebers and Feurer, 2016). Numerous studies have consistently reported significant EF impairment in MDD patients across multiple neuropsychological domains, including cognitive flexibility, inhibitory control, working memory, and task planning (Hack et al., 2023; Nuño et al., 2021; Snyder, 2013). The effective implementation of decision strategies requires the capacity to selectively focus attention and inhibit irrelevant stimuli, while consistent risk perception depends on the ability to flexibly shift between judgment contexts (Del Missier et al., 2010; Gerber et al., 2023). Notably, depression is associated with increased elaboration of negative information, difficulties in cognitive control when processing this information, and challenges disengaging from it (Kircanski et al., 2012; LeMoult and Gotlib, 2019). These manifestations reflect core deficits in cognitive control mechanisms, particularly in attentional regulation and emotional information processing (Keller et al., 2019). Depression often involves difficulties in controlling attention or regulating emotion, leading to challenges in disengaging from negative thoughts or threatening stimuli, thereby increasing negative biases in attention and memory and impeding top-down regulation of negative emotions (Yang et al., 2022). Given the connection between EF and metacognition, EF impairments associated with MDD, particularly within its core components, are likely to affect multiple metacognitive processes adversely.

In the current study, HCs exhibited a significantly higher drift rate in DCR + compared to DCR- conditions, which was absent in the patients. One possible explanation for the increased drift rate in the DCR + condition is related to the enhanced metacognitive control over decision process in DCR + condition. Drift rate reflects decision-making fluency: when the retrieval of information from decision-making is faster, the speed at which decision evidence accumulates and reaches the decision boundary should be higher (Ratcliff, 1978). In line with this view, the LMMs revealed that higher drift rate in the DCR + condition was associated with greater decision accuracy but shorter decision RTs, suggesting that efficient evidence accumulation facilitates both speed and precision in perceptual decision-making when metacognitive monitoring is engaged. Healthy individuals were capable of robust evaluations of their decisions and reported levels of confidence in their decisions that correlated with objective performance than MDD patients. These metacognitive abilities help them avoid making the same mistakes twice and prevent overcommitting time or resources to decisions that are based on unreliable evidence (Yeung and Summerfield, 2012). Confidence ratings may prompt them to allocate cognitive resources more efficiently, focusing their attention more selectively on the key information related to the decision and filtering out irrelevant distractions. As a result, cognitive processing becomes quicker and more efficient. In contrast, MDD patients did not show such a difference in drift rate between the DCR + and DCR- conditions. This could be attributed to several factors associated with depression. Therefore, depressive individuals have difficulty efficiently allocating cognitive resources and integrating decision-relevant information, which impairs their sensitivity to the evidence and leads to lower drift rate and decision accuracy. Depressive patients showed impaired metacognitive abilities and limited cognitive resources, which can influence their decision-making process. When constantly asked to provide confidence ratings about their decisions, it may exacerbate their sense of uncertainty and slow the accumulation of evidence. Similarly, Hauser et al. found that compulsivity spectrum disorders were associated with reduced metacognitive ability and perceptual decision-making deficits, where motion-related evidence accumulates more slowly (Hauser et al., 2017). Furthermore, previous research on decision-making in mental disorders has provided insights into the current findings. Among individuals with early psychosis, evidence accumulation is slower and less efficient, with less evidence utilized for decision-making and impaired integration of contextual information (Arend, 2024). Moreover, the present study aligned with previous DDM research on depression (Lawlor et al., 2020; Pitliya et al., 2022; Shen et al., 2024), revealing that depressive patients exhibit both conservative decision thresholds (wider boundaries) and suboptimal evidence accumulation rates (lower drift rates). The drift rate serves as a potential mechanism underlying the observed positive correlation between confidence and decision-making performance, where higher confidence levels correspond to improved processing efficiency and enhanced evidence accumulation (Hu et al., 2022; Liu and Lourenco, 2022). Hu et al. (2022) found that higher retrospective confidence ratings were associated with higher drift rates, further highlighting the critical interplay between metacognitive monitoring and decision efficiency.

We found longer RT in MDD patients than HCs, but no significant difference was observed when comparing non-decision time (*t*) between groups. In DDM, RTs can be decomposed into decision time and non-decision time, with the latter involving the time spent encoding and responding (Ratcliff and McKoon, 2008). The absence of group differences in non-decision time components suggested that the performance deficits in MDD originate specifically from impaired evidence accumulation processes rather than peripheral sensory encoding or motor execution stages.

This study has several limitations. First, limited evidence existed regarding the directionality and magnitude of reactivity effects associated with retrospective confidence ratings. While some studies found a negative or no reactivity effect of confidence ratings on decision accuracy (Ackerman et al., 2020; Baranski and Petrusic, 2001; Double and Birney, 2017, 2024), these inconsistencies may stem from variations in cognitive domains or experimental designs across studies. Future investigations should examine the reactivity effect across various cognitive domains to draw more specific conclusions. Further research is needed to elucidate whether individual differences in metacognitive ability influence the reactivity effect. Intuitively, if the reactivity effect represents the influence of metacognitive monitoring on online cognitive processing, individuals with better metacognitive ability should exhibit a more pronounced reactivity effect. Theoretically, if reactivity effects reflect metacognitive monitoring’s influence on online cognitive processing, individuals with superior metacognitive abilities should demonstrate more pronounced reactivity effects. Finally, the cross-sectional design and the potential effects of medication in the MDD patients preclude a comprehensive understanding of longitudinal trajectories in metacognitive dysfunction development among individuals with depression. Finally, this study has controlled the task difficulty around 70%. It is a common practice to keep a constant task performance to avoid confounding effect of first-level decision-making on reactivity effect. However, although evidence suggests that perceptual confidence rating may be independent of task performance (Dou et al., 2024), holding a certain level of task difficulty could affect confidence ratings and accuracy interpretations, and the interactions between confidence ratings and decision-making (reactivity effect). Further studies could allow the difficulty to vary in the task to test if the reactivity effect would be affected by task difficulty.

## 5 Conclusion

Our results suggested that the reactivity effect was impaired in patients with MDD. Unlike in HCs, eliciting decision confidence ratings failed to significantly enhance decision accuracy or evidence accumulation rate (drift rate) in MDD patients. The abnormal confidence reactivity effect highlighted compromised metacognitive control in MDD, which may underlie decision-making impairments through dysfunctional integration of confidence signals into cognitive processes.

## Data Availability

The raw data supporting the conclusions of this article will be made available by the corresponding authors upon reasonable request.
